# Doing exercise or sport together with one’s child is positively associated with mothers’ momentary affect in daily life, but not with higher levels of overall physical activity

**DOI:** 10.1186/s12889-020-08864-6

**Published:** 2020-05-19

**Authors:** Martina Kanning, Bridgette Do, Tyler B. Mason, Britni R. Belcher, Chih-Hsiang Yang, Genevieve F. Dunton

**Affiliations:** 1 Department of Sport Science, Chair of Social and Health Sciences, University of Konstanz, Universitätsstraße 10, 78464 Constance, Germany; 2 Department of Preventive Medicine, University of Southern California, Los Angeles, CA USA; 3 Departments of Preventive Medicine and Psychology, University of Southern California, Los Angeles, CA USA

**Keywords:** Ambulatory assessment, Ecological momentary assessment, Mood, Goal facilitation, Working mothers

## Abstract

**Background:**

Physical inactivity is a widespread problem with a great need for innovative intervention concepts to overcome it. Epidemiological studies have identified working women in high-income Western countries to be at greater risk for physical inactivity. The current study included working mothers and examined within-subject associations between doing exercise/sport together with one’s child and five different affective states, and with light physical activity (LPA) and moderate-to-vigorous physical activity (MVPA).

**Method:**

During 1 week, mothers (*N* = 192) completed up to eight ecological momentary assessment (EMA) surveys a day to assess momentary affect and certain situational circumstances (e.g., doing exercise/sport, being together with child). Physical activity was assessed objectively with waist-worn accelerometers.

**Results:**

Multilevel analysis showed that doing exercise/sport together with one’s child was associated with higher positive affect and lower negative affect compared to being active alone. However, greater frequency of doing exercise/sport together with children was negatively associated with MVPA.

**Conclusion:**

Due to the positive effect on momentary affect, combining spending time together with one’s child and simultaneously doing exercise/sport might be a good strategy of pairing two relevant personal goals. However, this strategy was not associated with sufficient MVPA.

## Background

Physical activity has well-established positive effects on physiological health, including decreasing risk for cardiovascular disease, hypertension, diabetes, and some cancers [1–3], as well as on mental health, including reducing depression and enhancing subjective wellbeing [4–6]. Given these beneficial health effects, the relative reduction of insufficient physical activity is one of the nine global targets to improve the prevention and treatment of non-communicable diseases [7]. The World Health Organization (WHO) recommends at least 150 min of aerobic physical activity with moderate intensity or 75 min of vigorous activities per week, or equivalent combinations [8]. Nevertheless, a recently published review of 358 population-based surveys from 168 countries stated that only 27.5% (95% CI 25.0–32.2) of the overall population met the WHO recommendations for aerobic physical activity [9]. Besides wide variations in inactivity prevalence between different regions and income groups, the pooled analysis identified that women were less physically active than men, with the greatest sex differences in high-income Western countries. In such countries, the majority of women work outside the home [10] and working mothers have been identified as a group at high risk for physical inactivity [11].

Mothers, especially of younger and of school-aged children may encounter competing demands as they attempt to balance childcare and work, which may increase barriers and challenges to participating in exercise or sport [12]. Because time is limited, these goals could conflict and mothers may have to decide between being physically active or spending time with their child, for instance. One possible solution is for mothers to engage in physical activity together with their child. According to intergoal facilitation, combining two relevant goals is associated with positive feelings given that there is both less competition between goals and goal progress [13]. Furthermore, mothers may attain sufficiently physical activity levels.

According to Diener et al. [14] goal progress is associated with positive emotions and psychological well-being: when people progress toward goals, they experience positive emotions and life satisfaction, and they experience less psychological distress. Recently, a meta-analysis of 85 studies found that successful goal pursuit was positively associated with subjective well-being with moderate-to-large effect-size [15]. However, less evidence exists on within-subject associations and on how goal progress is associated with well-being in everyday life. In two daily diary studies among married couples, effects of within-subject changes in goal progress on changes in affective states (e.g., feeling confident, happy, calm) were analyzed [16]. Daily goal progress predicted increases in positive affective states on both the same and on the following day. Also, a 20-day time-sampling study with 89 middle-aged adults assessed different strategies for managing the domains of work, family, and leisure [17]. Strategies that integrated goals from multiple domains were associated with higher overall subjective well-being (see also [17, 18]). Furthermore, a lot of evidence showed positive associations between physical activity and positive affective states in between-person relations (summarized in: [19]) and in within-person relations [20]. But only view studies analyzed how situational context factors like being physically active together or alone moderate this associations [21].

Based on the existing empirical evidence, the current study analyzed the interplay between physical activity and spending time together with one’s child and how it predicts affect throughout the day. Combining activity and being together with one’s child might allow mothers to attain more physical activity, and as a result increase their positive mood and well-being during everyday life. Thus, the purpose of the current analysis was to investigate if spending time together with one’s child or the combination of simultaneously doing exercise or sport while being together with one’s child was associated with momentary positive and negative affect in everyday life. Then, regression analyses estimated how doing exercise or sport together with one’s child predicted mothers’ overall physical activity.

## Methods

### Study participants

Participants included mothers enrolled in the Mothers and Their Children’s Health (MATCH) study. The MATCH study is a longitudinal observational study designed to examine the effects of maternal stress on their children’s obesity risk through real-time data capture methodologies [22]. Participants were recruited through informational flyers and in-person by study staff at elementary schools and community centers in the greater Los Angeles, California area. The inclusion criteria for the MATCH study were: (1) have a child that is between 8 and 12 years old, (2) have custody of the child for at least 50% of the time, and (3) able to read and speak English or Spanish. Study exclusion criteria included: (1) report a physical health condition that may prevent physical activity, (2) currently taking medications for thyroid functions or psychological conditions such as depression, anxiety, mood disorders, and ADHD (Attention Deficit Hyperactivity Disorder), (3) currently pregnant, (4) have a child that is currently enrolled in special education programs, (5) currently using oral or inhalant corticosteroids for asthma, (6) have a child that is considered underweight by a BMI percentile < 5%, and (7) work from home more than two weekday evenings per week (between the hours of 5-9 pm) or more than 8 h on any weekend day. Details on specific recruitment strategies and the protocol for the study are reported elsewhere [22].

A total of 202 women were enrolled in the MATCH study at baseline with their child. Ten women did not complete the ecological momentary assessment (EMA) surveys on the study smartphone, yielding 192 participants for the current analyses. Mothers’ ages ranged from 24 to 57 years old (*M* = 41.0, *SD* = 6.2), and the participating children’s ages ranged from 8 to 12 years old (*M* = 9.6, *SD* = 0.9). Participant characteristics at baseline are shown in Table 1.

**Table 1:** Participant characteristics (*N* = 202^a^)

Variable	*n* (%)
Working status^b^	
Work full-time	114 (56.6)
Work part-time	51 (25.8)
Other	33 (16.7)
Child sex	
Male	99 (49.0)
Female	103 (51.0)
Mother race/ethnicity^b^	
Hispanic/Latino	99 (49.0)
Not Hispanic/Latino	101 (50.0)
Child race/ethnicity^b^	
Hispanic/Latino	114 (56.4)
Not Hispanic/Latino	86 (42.6)
Education level^b^	
Not college graduate	79 (39.1)
College graduate	117 (57.9)
Type of household^b^	
Single parent	46 (22.8)
Non-single parent	155 (76.7)
Annual household income	
Less than $34,999	56 (27.7)
$35,000–$74,999	49 (24.3)
$75,000–$104,999	44 (21.8)
Greater than $105,000	53 (26.2)

^a^Data from all participants enrolled at baseline; ^b^Datamissing on variable

### Procedure

Eligible participants attended two in-person data collection sessions. During the baseline session, participants provided written informed consent. Participants also completed paper-pencil questionnaires. Mothers were loaned an Android smartphone (MotoG or Motorola X) with a data plan for the study monitoring period (Motorola Mobility, Chicago, IL). EMA data were collected through a custom software phone application for smartphones utilizing an Android operating system (Google Inc., Mountainview, CA). The study app was available in English or Spanish. Participants were trained on how to use the study application through both verbal and written instructions. EMA data were wirelessly uploaded and stored on a secure internet-accessible server.

The monitoring period lasted a total of six complete days and two partial-days. During the monitoring period, mothers were asked to carry the study smartphone with them while they were awake, aside from non-compatible activities (i.e., sleeping, showering, and swimming). Participants were instructed to connect the study phone to their home wireless Internet (Wi-Fi). If wireless connection was not available, EMA data were downloaded directly from the study phone when it was returned to the research staff.

Participants completed signal-contingent EMA surveys via the phone application through a stratified sampling scheme. Mothers were prompted up to four times per day on weekdays and up to eight times per day on weekend days. On weekdays, participants were prompted randomly once during each of the following time windows: 3:00–4:00 P.M., 5:00–6:00 P.M., 7:00–8:00 P.M., and 9:00–9:30 P.M. On weekend days, participants were prompted randomly once during each of the following time windows: 7:00–8:00 A.M., 9:00–10:00 A.M., 11:00 A.M.-12:00 P.M., 1:00–2:00 P.M. 3:00–4:00 P.M., 5:00–6:00 P.M., 7:00–8:00 P.M., and 9:00–9:30 P.M.

Participants reported their sleep and wake-up times to ensure that surveys were not prompted when they were asleep. Participants were notified to complete surveys through an audible prompt and/or vibration. On average, each survey took about 2 min to complete [23]. If no entry was made, the application sent up to two reminder signals at 3-min intervals. After the final reminder, the EMA survey became inaccessible to the participant. Participants were instructed to ignore signals during any inconvenient activities, such as driving. During the second data collection session, participants returned all study materials and were compensated. This study was conducted in accordance with the Declaration of Helsinki and all aspects of the study were approved by the Institutional Review Boards at the University of Southern California and Northeastern University (HS-12-00446).

### Measures

#### Participant characteristics

Participants reported demographics and personal characteristics through a paper-pencil questionnaire. Demographics such as age and race/ethnicity were collected for both the mother and their participating child. In addition, participants reported their self-rated health through a single item [24], their household status (single parent household vs. not a single-parent household), and their working status. The current analyses controlled for whether the family household was not a single-parent household (0) or single-parent household (1), and whether mothers were working employed full-time (0) or employed part-time (1).

#### Affective states

Five affective states were based on the Positive and Negative Affect Schedule (PANAS: [25, 26]) were assessed. Each affective state was measured by the following items, respectively: “Right before the phone went off, how (1) happy, (2) calm, (3) stressed, (4) angry, and (5) sad/depressed were you feeling?” The response options consisted of a scale ranging from 1 (*Not at all*) to 4 (*Extremely*).

#### Global life satisfaction

Self-reported global life satisfaction was measured through the Satisfaction with Life Scale (SWLS: [27]) via a paper-pencil questionnaire given during the baseline session. The five-item measure includes items such as “In most ways my life is close to ideal” and “I am satisfied with my life” with a 7-point response scale ranging from 1 (*Strongly disagree*) to 7 (*Strongly agree*). A mean score was computed for the five items, with higher scores indicating higher satisfaction. The Cronbach’s alpha among the study sample was .90, indicating high internal consistency.

#### Objective physical activity

For the study monitoring period, continuous objective physical activity counts were collected through accelerometry. The waist-worn accelerometer (Actigraph GT3X) used 30-s epochs to collect body movement in activity counts. Intervals with ≥60 continuous minutes of zero activity counts were considered as device non-wear. Only data with valid days (> 10 h of device wear) was used for the analyses. Accelerometer data were time-stamped in order to be matched with the EMA responses. For the within-subject analyses, the total number of physical activity counts in the 120 min leading up to an EMA prompt was used. To control for valid device wear time, only intervals with ≥80 min valid device wear time within this period were included in the analyses. To estimate how doing exercise or sport together with one’s child predicted mother’s overall physical activity, the overall averaged minutes per day of light physical activity (LPA) and moderate to vigorous activity (MVPA) were derived used established cut-points [28] and used in the regression analyses.

#### Time spent with child and doing sport or exercise with child

During each EMA prompt, participants indicated whether they had spent time with their child by responding to the following question: “Over the last 2 hours, have you spent time with your child (together in the same location)?” Response options were scored as 1 (*Yes*) and 0 (*No*). In addition, self-report physical activity was measured by the item “Over the last 2 hours, which of these things have you done? (check all)”. Participants were given the option to select “Exercise or sports”. If participants selected “Exercise or sports”, the study application automatically asked the participant “Was anyone with you when you were doing exercise or sports? (check all). Response options included 1 (*No (alone)*)”, 2 (*My child)*, 3 (*Spouse/romantic partner*), and 4 (*Othe*r). For the current analyses, a new variable was created to indicate whether the mother did exercise or sport alone (0) or with the child (1).

### Data analyses

We conducted two sets of multilevel random coefficient regression models to investigate the within-subject effects of spending time together with one’s child (*together with child*) (model 1a-e) and doing sport or exercise together with one’s child (*sport together with child*) (model 2a-e) on each on five momentary affective states (happy [model 1a and 2a], calm [model 1b and 2b], stressed [model 1c and 2c], angry [model 1d and 2d], and sad/depressed [model 1e and 2e]). We nested repeated prompts (level-1) within mothers (level-2) to analyze the aforementioned associations and to control for the sum of 120 min of physical activity counts prior to the prompt (PA) (level-1), general life-satisfaction (SWLS), and working status (WS) (both level-2). Restricted maximum likelihood estimations were used and analyses were conducted in SPSS 25. The alpha-level was set at *p* < 0.05. Intra-class correlations for each dependent affect measure were estimated.

The equations for the hierarchical levels are as follows:

Model 1 a-e

1 $$ \mathrm{Level}\ 1:{\mathrm{affect}}_{\mathrm{ti}}={\mathrm{b}}_0\ \left({\mathrm{constant}}_{\mathrm{ti}}\right)+{\mathrm{b}}_1\ \left({\mathrm{PA}}_{\mathrm{ti}}\right)+\mathrm{b}2\ \left( together\ {with\ child}_{\mathrm{ti}}\right)+{\mathrm{r}}_{\mathrm{ti}} $$

2 $$ \mathrm{Level}\ 2:{b}_0={\gamma}_{0 0}+{\gamma}_{0 1}\ (SWLS)+{\gamma}_{0 2}\ (WS)+{\mu}_{0 i} $$

Model 2 a-e

3 $$ \mathrm{Level}\ 1:{\mathrm{affect}}_{\mathrm{ti}}={\mathrm{b}}_0\ \left({\mathrm{constant}}_{\mathrm{ti}}\right)+{\mathrm{b}}_1\ \left({\mathrm{PA}}_{\mathrm{ti}}\right)+\mathrm{b}2\ \left( sport\ together\ {with\ child}_{\mathrm{ti}}\right)+{\mathrm{r}}_{\mathrm{ti}} $$

4 $$ \mathrm{Level}\ 2:{b}_0={\gamma}_{0 0}+{\gamma}_{0 1}\ (SWLS)+{\gamma}_{0 2}\ (WS)+{\mu}_0 $$

Equation 1 represents the subject’s response (subscript _i_) for one of the five affect measures (affect_*ti*_) for any given EMA entry (subscript _t_). Affect_*ti*_ is calculated as the average intercept of one affect measure across all subjects (*b*_*0*_) and two level-1 predictors: the sum of 120 min of physical activity counts prior diary entry (b_1_ (PA _ti_)) and time spending with one’s child during the last 120 min (b2 (*together with child*_ti_)) in Model 1 a-e and doing exercise or sport together with one’s child during the last 120 min (b2 (*sport together with child*_ti_)) in Model 2 a-e. Physical activity (PA) was mean-centered by groups, where the group refers to a person. In doing this, we are able to disaggregate the between- and the within-subject effects [29]. Both dichotomous variables (*together with child*, *sport together with child*) were not group-centered and used in its natural metric: together with child (1), doing exercise or sport together (1), and not together with child (0), and not doing exercise or sport together with child (0). Even though a predictor in its natural metric includes within- and between-person effects, this allows estimation of exact increases in affective states when the mother is together with child or doing exercise or sport together with child versus not. Random effects for each level-1 predictor were added; however, we only retained significant random effects in the final model. The random effect for the level-1 model is given by *r*_*ti*_, which is assumed to have a normal distribution, “0” mean, and a variance of σ^2^.

To control for between-subject effects, two level-2 predictors were added. Thus, this level included the fixed effects, γ of the average intercepts and slopes across all subjects, two covariates general satisfaction with life (*SWLS*) and working status (WS), as well as the random effects (*μ*_0i,_). General satisfaction with life was grand mean-centered while working status was used in its natural metric. The random effects are assumed to be multivariate and normally distributed, with both having expected values of “0”.

To clarify the magnitude of significant effects, we calculated standardized effects for variables with interval scale (i.e., *PA* and *SWLS*) and effect size *r* for dichotomous variables (i.e., *together with child*, *sport together with child, and WS*). To calculate standardized effects for momentary volume of daily physical activity and general satisfaction with life (eq. 5), the standard deviation was obtained from the sample mean of total activity counts of the last 120 min before each EMA entry or the sample mean of general satisfaction with life and from the averaged within-subject mean of the corresponding affect subscale.

5 $$ \mathrm{Standardized}\ \mathrm{effect}={b}_{1\mathrm{i}}\ast \mathrm{SD}\ \left( PA\ \mathrm{or}\  SWLS\right)/\mathrm{SD}\ \left(\mathrm{correspondent}\ \mathrm{affect}\ \mathrm{measure}\right) $$

To reveal the differences between each of the two characteristic attributes (1 vs 0) of the three dichotomous variables, we used effective degrees of freedom to estimate effect sizes considering the nested structure of the data (Nijders and Bosker, 2011).

6 $$ {N}_{\mathrm{effective}}= Nn/\left(1+\left(n-1\right)\ast {\uprho}_{\mathrm{I}}\right) $$

N*n* indicates the number of measurement points, *n* stands for the average number of measurement points per person, and ρ_Ι_ represents the intra-class coefficient of the corresponding affect measure (feeling happy, calm, stressed, angry, and sad/depressed). Effective degrees of freedom were analyzed with *N*_effective_ minus the number of predictors. We calculated effect size *r*, using *t*-values and effective degrees of freedom.

To estimate the extent to which the frequency of doing exercise or sport together with one’s child predicted overall LPA and MVPA (minutes/day), we averaged the dichotomous variable *sport together with child* per person (mother). Multiple regression analyses were used to estimate if this aggregated variable (sport together with child averaged per mother), as well as several demographics and covariates (e.g., mother or child age, child sex, mother education level, mother or child race/ethnicity, household type, self-rated health), predicted overall LPA and MVPA (minutes/day).

## Results

### Descriptive statistics

Over the entire assessment period, the 192 mothers received 5818 EMA prompts, of which they completed 4445 prompts (76%). On average, mothers completed 23 EMA surveys (*SD* = 7.6) with a range from 1 to 35. Descriptive statistics for affect and physical activity are reported in Table 2. The results of the intra-class coefficients were ρ_I_ = 0.31, ρ_I_ = 0.37, ρ_I_ = 0.38, ρ_I_ = 0.5, and ρ_I_ = 0.33 for happy, calm, stressed, angry, and sad/depressed, respectively, indicating that 69, 63, 62, 50, and 67% of the respective affect measure variance was caused by within-subject variation (e.g., due to situational effects).

**Table 2:** Descriptive statistics of the five affect measures

Variable	Between-subject		Within-subject		Min	Max
	Mean	SD	Mean	SD		
Feeling happy	2.75	0.48	2.75	0.80	1	4
Feeling calm	2.54	0.48	2.54	0.89	1	4
Feeling stressed	1.55	0.37	1.56	0.72	1	4
Feeling angry	1.37	0.27	1.37	0.66	1	4
Feeling sad/depressed	1.17	0.26	1.17	0.46	1	4

After removing 64 outlier situations (PA > Mean + 2.5 SD), participants recorded on average 27,512 counts in the 120-min window prior to the EMA prompts (*SD* = 17,933, median = 23,741, range = 17–103,982). Overall average minutes per day of LPA was 198.52(*SD* = 65.95, median = 194.56, range = 29.06 to 397.75). LPA was normally distributed. Overall average minutes per day of MVPA was 21.35 (*SD* = 15.18; median = 17.62, range = 1.56–86.56). MVPA was log-transformed because it was not normally distributed.

In 3525 situations (79%), mothers reported being together with their child in the same location during the last 2 hours (*together with child*). In 464 situations (11%), mothers indicated that during the last 2 hours they engaged in sport or exercise. In 43% (201) of these sport or exercise situations, they did exercise or sport together with their child, and in 171 of these situations (36%), they did exercise or sport alone. In the remaining situations (21%), they did exercise or sport with another person (e.g., spouse or romantic partner). For the regression analyses, the situation-specific variable *sport together with child* was aggregated for each mother. This aggregated variable ranged from 0 (no situation in which the mother did exercise or sport with her child, 25%) to 1 (in all situations mothers did exercise or sport with her child, 41%) and had a mean of 0.57 (*SD* = 0.42, median = 0.62).

The fixed and random effects for each of the affect measures are displayed in Table 3. Random error terms of some slopes were not significant in all models and had to be fixed because the random and the fixed variability of those slopes cannot be reliably separated. This implies that the fixed effects may vary, but they do not vary randomly as a function of general satisfaction with life and working status.

**Table 3:** Fixed and random effects and variance components for happy (model 1a & 2a), calm (model 1b & 2b), stressed (model 1c & 2c), angry (model 1d & 2d), and sad/depressed (model 1e & 2e)

Outcome	Fixed					Random					
Predictor	Coefficient	SE	t-Value	df	***p***-Value	Coefficient	SD	Wald Z	***p***-value	95%-Predictive Interval ^**a**^	
**Feeling Happy**											
Model 1a (participants: *N* = 183; prompts: *n* = 3270)											
Intercept	2.578	0.076	33.873	2428	< .001	0.168	0.020	8.045	< .001	0.13	0.21
SWLS	0.157	0.024	6.408	173	< .001						
Work Status	0.057	0.033	1.710	3301	.087						
PA (120 min)	8.9E-7	6.7E-7	1.331	3224	.183						
Together with child	0.082	0.031	2.653	3356	.008						
Model 2a (participants: *N* = 103; prompts: *n* = 249)											
Intercept	2.829	0.195	14.472	264	< .001	0.215	0.050	4.251	< .001	0.14	0.34
SWLS	0.115	0.045	2.427	113	.013						
Work Status	−0.069	0.099	−0.697	240	.486						
PA (120 min)	2.17E-6	1,69E-6	1.283	245	.201						
Sport to-gether with child	0.377	0.086	4.353	273	< .001						
**Feeling Calm**											
Model 1b (paricipants: *N* = 182; prompts: *n* = 3296)											
Intercept	2.34	0.086	27.051	2812	< .001	0.161	0.021	7.719	< .001	0.13	0.21
SWLS	0.163	0.024	6.635	175	< .001						
Work Status	0.071	0.038	1.834	3307	.067						
PA (120 min)	−3.0E-6	7.83E-7	−3.838	3228	< .001						
Together with child	0.085	0.035	2.551	3380	.018						
Model 2b (participants: *N* = 103; prompts: *n* = 249)											
Intercept	2.137	0.216	9.888	262	< .001	0.289	0.067	4.280	< .001	0.18	0.45
SWLS	0.180	0.051	3.465	108	< .001						
Work Status	0.113	0.110	1.030	234	.304						
PA (120 min)	−1.8E-7	1,8E-6	−0.098	239	.922						
Sport to-gether with child	0.388	0.096	4.039	271	< .001						
**Feeling stressed**											
Model 1c (participants: N = 183; prompts: *n* = 3268)											
Intercept	1.810	0.072	25.127	2848	< .001	0.106	0.014	7.525	< .001	0.08	0.13
SWLS	−0.085	0.020	−4.051	170	< .001						
Work Status	−0.087	0.032	−2.666	3304	.008						
PA (120 min)	6.8E-7	6.5E-7	1.046	3223	.296						
Together with child	−0.106	0.030	−3.552	3382	< .001	0.023	0.011	2.081	.037	0.01	0.06
Model 2c (participants: *N* = 103; prompts: *n* = 249)											
Intercept	1.915	0.175	10.890	268	< .001	0.107	0.034	3.101	.002	0.06	0.20
SWLS	−0.07	0.036	−1.885	99	.062						
Work Status	−0.152	0.090	−1.673	250	.095						
PA (120 min)	1.6E-6	1.6E-6	−0.168	315	.313						
Sport to-gether with child	−0.311	0.077	−4.037	277	< .001						
**Feeling Angry**											
Model 1d (participants: *N* = 183; prompts: *n* = 3267)											
Intercept	1.430	0.070	20.332	3323	< .001	0.051	0.008	6.353	< .001	0.03	0.07
SWLS	−0.066	0.015	−4.362	161	< .001						
Work Status	−0.032	0.032	−1.015	3228	.310						
PA (120 min)	1.1E-6	6.3E-7	1.680	3139	.093						
Together with child	−0.001	0.028	−0.041	3322	.968						
**Model 2d** (participants: *N* = 103; prompts: *n* = 249)											
Intercept	1.625	0.206	7.864	270	< .001	0.048	0.025	1.918	.05	.02	.13
SWLS	−0.094	0.032	−2.937	94	.004						
Work Status	−0.090	0.016	−0.852	.268	395						
PA (120 min)	−8.4E-7	1.6E-6	−0.520	264	.604						
Sport to-gether with child	−0.201	0.075	−2.668	265	.008						
**Feeling sad/depressed**											
Model 1e (participants: *N* = 183; prompts: *n* = 3270)											
Intercept	1.224	0.045	26.678	2505	< .001	0.056	0.007	8.023	< .001	.04	.07
SWLS	−0.076	0.014	−5.352	173	< .001						
Work Status	−0.017	0.020	−0.870	3222	.385						
PA (120 min)	1,5 E-7	3.9 E-7	0.402	3143	.402						
Together with child	−0.021	0.018	−1.152	3274	.249						
**Model 2e** (participants: *N* = 103; prompts: *n* = 249)											
Intercept	1.299	0.126	10.275	265	< .001	0.050	0.012	4.054	< .001	.03	.08
SWLS	−0.127	0.023	−5.408	123	< .001						
Work Status	−0.054	0.065	−0.828	259	.409						
PA (120 min)	3.6 E-7	9.9 E-7	0.369	248	.712						
Sport to-gether with child	−0.116	0.047	−2.460	268	.015						

^a^Based on the assumption of normally distributed regression coefficients, the 95% predictive interval indicates the range of values between which 95% of the regression coefficients are estimated to lie (Hox, 2010). The intervals were calculated based on a model without Level 2 predictors

### Together with one’s child in the same location

In model 1a-e, we analyzed how five different affective states were predicted by being together with one’s child (or not) over the past 120 min (see Table 3). In situations of being together with one’s child, mothers felt happier (effect size *r* = 0.13), felt calmer (effect size *r* = 0.13), and felt less stressed (effect size *r* = 0.19) compared to situations of not being together. Greater averaged counts of objectively measured PA during the prior 120 min predicted less calmness (standardized effect = − 0.01). Furthermore, general life satisfaction was significantly associated with all affective states. According to the standardized effects, if satisfaction with life increased by 1 SD, feeling happy increased by 0.96, feeling calm increased by 0.90, feeling stressed decreased by 0.58, feeling angry decreased by 0.49, and feelings of sadness and depression decreased by 0.81 across all EMA prompts. In the model predicting feeling stressed, working status was negatively associated: mothers with a part-time job experienced more feelings of stress than mothers with a full-time job (effect size *r* = 0.14).

### Doing exercise or sport together with one’s child

In model 2a-e (see Table 3), we exchanged the variable *together with child* with *sport together with child* to assess the effect of being together and simultaneously doing exercise or sport together with one’s child (or not) over the past 120 min and five affective states. Doing exercise or sport together with one’s child over the past 120 min was a significant predictor in all affect models, suggesting that when a mother did sport or exercise together with her child, she felt happier (effect size *r* = 0.64), felt calmer (effect size *r* = 0.64), felt less stress (effect size *r* = 0.71), felt less angry (effect size *r* = 0.56), and felt reduced sad/depressed (effect size *r* = 0.43) compared to EMA prompts when she did exercise or sport alone. This effect was significant above and beyond the (non-significant) effects of the averaged counts of physical activity during the same time period of the prior 120 min. Similar to the models 1a-e, general satisfaction with life was positively associated with feeling happy and calm, and negatively associated with feeling angry and sad/depressed with no relevant changes in effect sizes. In contrast to model 1c, feeling stressed was not significantly associated with satisfaction with life (*p* = .06). Working status did not significantly predict any affective states in these models (2a-e).

### Prediction of mothers overall physical activity

Two multiple regression analyses were performed to predict overall LPA and overall MVPA. Both regression analyses were significant (LPA: F _(9)_ = 2.08, *p* = .037; MVPA: F _(9)_ = 2.83, *p* = .005) explaining 15% variance for LPA and 19% variance for MVPA. The frequency of doing exercise or sport together with children significantly predicted MVPA (β = −.24, *p* = .008). The more mothers reported doing exercise or sports alone during the assessment period, the more they engaged in MVPA. Self-rated health was significant in both models (LPA: β = .21, *p* = .027; MVPA: β = .21, *p* = .039), indicating that mothers who rated their general health as better also engaged in more LPA or MVPA.

## Discussion

This ambulatory assessment study among 192 mothers sought to determine whether spending time together with one’s child or the combination of spending time together and simultaneously doing exercise or sport together was positively associated with five momentary affective states in everyday life. In addition, the extent to which mothers do exercise or sport with their child was examined as a predictor of overall LPA and MVPA.

Results of the within-subject associations showed that mothers felt happier, calmer, and less stressed when they were with their child in daily life. This result is consistent with cross-sectional studies and theoretical assumptions about the important role of close relationships in fostering well-being and quality of life [30]. However, this study extended existing research by providing empirical evidence for within-subject relations in mother’s daily life. In addition to spending time together with one’s child or not, physical activity was associated with affective states: mothers who were more physically active than usual during this time period reported less calmness. There were no significant associations between doing more physical activity than usual and feelings of happiness or other negative affective states (e.g., feeling angry, sad). This pattern of findings is partly in line with other within-subject analysis of the relation between physical activity and affective states in daily life [20, 31]. In a review of the effects of physical activity in daily life on subsequent affective states, most studies reported evidence of associations between more positive affect and greater physical activity, whereas there were inconsistent effects for physical activity decreasing subsequent negative affect [20]. Furthermore, most evidence suggested that physical activity increased feelings of energy rather than feelings of happiness. However, effects on calmness were inconsistent; some studies showed that people do not feel relaxed and calm while being physically active. People might feel more relaxed, but only after a time delay when the direct exhaustion of being active passes [32].

In addition to spending time together with one’s child, simultaneously doing sport or exercise together was associated with more positive affect and lower negative affect. This finding expands existing results because we used this EMA study to analyze moderating effects of situational circumstances on the association between affective states and physical activity in daily life. According to the result, doing sport or exercise together with one’s child positively moderated the association between physical activity and affective states. Although this study did not analyze personal goals directly, the results may be explained by the possibility that doing sport and exercise with one’s child involves the facilitation of dual goals (i.e., exercising and spending time with one’s child), which may increase positive affect. Our findings are in line with former studies about the effects of goal facilitation on subjective wellbeing, although these former studies analyzed other combinations of two goals (e.g. inter-domain relationships across work and family) [33]. In an EMA study analyzing within-subject relations [17], participants reported how regular daily activities (e.g., reading, eating, talking) facilitated personal goals and their current affective state. Participants felt better and reported higher goal facilitation in situations where they did something that concerned more than one life domain.

### Effects of exercise or sport with children on LPA and MVPA

Mothers had more MVPA minutes when they were physically active alone. Thus, although doing exercise or sport together with one’s child was associated with better momentary feelings, it was also negatively associated with overall MVPA. Mothers had more MVPA minutes when they were physically active alone. One possible explanation could be, that doing exercise or sport together with the child may include activities that are more intermittent or of lower intensity than the MVPA threshold. Furthermore, mother’s attention may focus on watching their kid(s) and ensure their safety, instead of fully engage in sports and exercising for their own benefits, compared to when the exercise alone. Also, mothers who are not frequently engaging in exercise or sport with her child may have other strategies in her daily life to integrate MVPA (e.g., active transportation or active housework).

### Limitations and future directions

Although this study was one of the first to assess within-subject variations of affective states associated with spending time together with one’s child and doing exercise or sport together with one’s child, there are several limitations worth noting. First, we did not explicitly assess if spending time with one’s child or being sufficiently physically active were relevant personal goals to mothers. The majority of mothers reported primarily doing exercise or sport together with their child (41%), while 25% reported almost never doing exercise or sport with their child and the remaining third varied across the EMA period. However, this study did not explicitly assess if this 41% of mothers had the goal to do exercise or sport together with their child. While some mothers may have goals to spend time together with their child while doing exercise or sport, others may do exercise and sport to reach other personal goals. Mothers might have personal goals for activity (e.g., having fun, meeting friends, experience), but those goals may not allow them to meet the WHO-recommendation for being sufficiently physically active. Future studies should evaluate the extent to which being together with one’s child and doing exercise or sport are personally relevant goals for mothers. Furthermore, there is a need for more research analyzing the effect of goal facilitation on the performance of physical activity. Doing exercise or sport, which may simultaneously lead to progress in another relevant goal, might support mothers in achieving recommended physical activity levels.

Another limitation of the study is that the waist-worn accelerometer is not an ideal measurement to capture PA from cycling [34] and because our accelerometer was not waterproof, it was not able to access swimming activities [34]. These activities may be common for mothers and children to engage in unstructured activities during leisure time together [35]. A further limitation is that participants might have understood the daily-diary question about exercise and sport in different ways. Although all participants received a definition which activities fit to this question, there could be between person differences in rating everyday life activities like walking correctly.

In addition, the current analyses did not differentiate between weekend and weekday patterns; weekends provide more opportunities for doing exercise or sport together.

## Conclusions

This study showed that mothers of 8 to 12-year-old children experienced happier and calmer feelings and reduced stress in daily life when they were with their children. This positive effect on momentary affective states remained when mothers did exercise or sport with their child and was also associated with reduced feelings of anger and sadness. Therefore, doing exercise or sport together with one’s child might be a good strategy of pairing two goals relevant to spending time with children and health. Furthermore, doing exercise or sport together with one’s child may be a useful health promotion strategy for increasing physical activity given that positive affect can increase physical activity [36]. However, in this study, combining spending time together with one’s child and doing exercise or sport was negatively associated with physical activity.

## Data Availability

The datasets used and/or analysed during the current study are available from the corresponding author on reasonable request.

## Abbreviations

LPA: Light physical activity
MVPA: Moderate-to-vigorous physical activity EMA Ecological momentary assessment
